# Supplementary material for: SeqRepo: A system for managing local collections of biological sequences
Source: PLoS One. 2020 Dec 3;15(12):e0239883. doi: 10.1371/journal.pone.0239883 (PMC7714221; doi:10.1371/journal.pone.0239883)
Supplement: S1 Code — (PDF) [file pone.0239883.s001.pdf]

Truncated Digest Collision Analysis

September 13, 2020

1 Truncated Digest Timing and Collision Analysis

SeqRepo assigns identifiers to sequences based on a SHA-512 digest, truncated to 24 bytes (192 bits), and `base64url` [8] encoded. This notebook discusses the choice of SHA-512 over other digest methods and the choice of truncation length. This scheme is also used in the GA4GH Computed Identifiers and by the RefGet reference sequence protocol.

Source: Reece Hart, CC-BY

1.1 Conclusions

- The computational time for SHA-512 is similar to that of other digest methods on 64-bit CPUs, and is believed to be at least as cryptographically robust as other methods tested.
- 24 bytes (192 bits) of digest should be ample. For example, the probability of collision for a 24-byte digest over 1e18 messages is less than 1e-21.

[1]:
```
import hashlib
import math
import timeit

from IPython.display import display, Markdown

from utils import _format_time

algorithms = {'sha512', 'sha1', 'sha256', 'md5', 'sha224', 'sha384'}
```

1.2 Digest Timing

This section provides a rationale for the selection of SHA-512 as the basis for the Truncated Digest.

[2]:
```
def blob(l):
    """return binary blob of length l (POSIX only)"""
    return open("/dev/urandom", "rb").read(l)

def digest(alg, blob):
    md = hashlib.new(alg)
    md.update(blob)
```

```
    return md.digest()

def magic_run1(alg, blob):
    t = %timeit -o digest(alg, blob)
    return t

def magic_tfmt(t):
    """format TimeitResult for table"""
    return "{a} ± {s} ([{b}, {w}])".format(
        a = _format_time(t.average),
        s = _format_time(t.stdev),
        b = _format_time(t.best),
        w = _format_time(t.worst),
    )
```

[3]:
```
blob_lengths = [100, 1000, 10000, 100000, 1000000]
blobs = [blob(l) for l in blob_lengths]
```

[4]:
```
table_rows = []
table_rows += [["algorithm"] + list(map(str,blob_lengths))]
table_rows += [["-"] * len(table_rows[0])]
for alg in sorted(algorithms):
    r = [alg]
    for i in range(len(blobs)):
        blob = blobs[i]
        t = timeit.timeit(stmt='digest(alg, blob)', setup='from __main__ import␣
↪alg, blob, digest', number=1000)
        r += [_format_time(t)]
    table_rows += [r]
table = "\n".join(["|".join(map(str,row)) for row in table_rows])
display(Markdown(table))
```

algorithm	100	1000	10000	100000	1000000
md5	1.41 ms	2.99 ms	21.8 ms	157 ms	1.57 s
sha1	1.01 ms	2.02 ms	13.8 ms	123 ms	1.24 s
sha224	1.23 ms	3.46 ms	28.9 ms	273 ms	2.53 s
sha256	1.12 ms	3.25 ms	28.1 ms	272 ms	2.56 s
sha384	1.17 ms	2.57 ms	18.9 ms	178 ms	1.61 s
sha512	1.06 ms	2.6 ms	16.9 ms	178 ms	1.77 s

Conclusion: SHA-512 computational time is similar to that of other digest methods.

This is result was not expected initially. On further research, there is a clear explanation: The SHA-2 series of digests (which includes SHA-224, SHA-256, SHA-384, and SHA-512) is defined using 64-bit operations. When an implementation is optimized for 64-bit systems (as used for these timings), the number of cycles is essentially halved when compared to 32-bit systems and digests

that use 32-bit operations. SHA-2 digests are indeed much slower than SHA-1 and MD5 on 32-bit systems, but such legacy platforms is not relevant to the Truncated Digest.

1.3 Collision Analysis

Our question: **For a hash function that generates digests of length b (bits) and a corpus of m messages, what is the probability p that there exists at least one collision?** This is the so-called Birthday Problem [6].

Because analyzing digest collision probabilities typically involve choices of mathematical approximations, multiple "answers" appear online. This section provides a quick review of prior work and extends these discussions by focusing the choice of digest length for a desired collision probability and corpus size.

Throughout the following, we'll use these variables:

- P = Probability of collision
- P' = Probability of no collision
- b = digest size, in bits
- s = digest space size, $s = 2^b$
- m = number of messages in corpus

The length of individual messages is irrelevant.

1.3.1 Background: The Birthday Problem

Directly computing the probability of one or more collisions, P, in a corpus is difficult. Instead, we first seek to solve for P', the probability that a collision does not exist (i.e., that the digests are unique). Because are only two outcomes, $P + P' = 1$ or, equivalently, $P = 1 - P'$.

For a corpus of size $m = 1$, the probabability that the digests of all $m = 1$ messages are unique is (trivially) 1:

$$P' = s/s = 1$$

because there are s ways to choose the first digest from among s possible values without a collision.

For a corpus of size $m = 2$, the probabability that the digests of all $m = 2$ messages are unique is:

$$P' = 1 \times (\frac{s-1}{s})$$

because there are $s - 1$ ways to choose the second digest from among s possible values without a collision.

Continuing this logic, we have:

$$P' = \prod_{i=0}^{m-1} \frac{(s-i)}{s}$$

or, equivalently,

$$P' = \frac{s!}{s^m \cdot (s-m)!}$$

When the size of the corpus becomes greater than the size of the digest space, the probability of uniques is zero by the pigeonhole principle. Formally, the above equation becomes:

$$P' = \begin{cases} 1 & \text{if } m = 0 \\ \prod_{i=0}^{m-1} \frac{(s-i)}{s} & \text{if } 1 \leq m \leq s \\ 0 & \text{if } m > s \end{cases}$$

For the remainder of this section, we'll focus on the case where $1 \leq m \ll s$. In addition, notice that the brute force computation is not feasible in practice because m and s will be very large (both $\gg 2^9$).

1.3.2 Approximation #1: Taylor approximation of terms of P'

The Taylor series expansion of the exponential function is

$$e^x = 1 + x + \frac{x^2}{2!} + \frac{x^3}{3!} + ...$$

For $|x| \ll 1$, the expansion is dominated by the first terms and therecore $e^x \approx 1 + x$.

In the above expression for P', note that the product term $(s-i)/s$ is equivalent to $1 - i/s$. Combining this with the Taylor expansion, where $x = -i/s$ ($m \ll s$):

$$\begin{aligned} P' &\approx \prod_{i=0}^{m-1} e^{-i/s} \\ &= e^{-m(m-1)/2s} \end{aligned}$$

(The latter equivalence comes from converting the product of exponents to a single exponent of a summation of $-i/s$ terms, factoring out $1/s$, and using the series sum equivalence $\sum_{j=0}^{n} j = n(n+1)/2$ for $n \geq 0$.)

1.3.3 Apprixmation #2: Taylor approximation of P'

The above result for P' is also amenable to Taylor approximation. Setting $x = -m(m-1)/2s$, we continue from the previous derivation:

$$\begin{aligned} P' &\approx e^{-(m(m-1)/2s} \\ &\approx 1 + \frac{-m(m-1)}{2s} \end{aligned}$$

1.3.4 Approximation #3: Square approximation

For large m, we can approximate $m(m-1)$ as m^2 to yield

$$P' \approx 1 - m^2/2s$$

.

1.3.5 Summary of equations

We may now summarize equations to approximate the probability of digest collisions.

Method	Probability of uniqueness(P')	Probability of collision($P = 1 - P'$)	Assumptions	Source/Comparison
exact	$\prod_{i=0}^{m-1} \frac{(s-i)}{s}$	$1 - P'$	$1 \leq m \leq s$	[6]
Taylor approximation #1	$e^{-m(m-1)/2s}$	$1 - P'$	$m \ll s$	[6]
Taylor approximation #2	$1 - \frac{m(m-1)}{2s}$	$\frac{m(m-1)}{2s}$	(same)	[6]
Large square approximation	$1 - \frac{m^2}{2s}$	$\frac{m^2}{2s}$	(same)	[5](where $s = 2^n$)

1.4 Choosing a digest size

Now, we turn the problem around: **What digest length b corresponds with a collision probability less than P for m messages?**

From the above summary, we have $P = m^2/2s$ for $m \ll s$. Rewriting with $s = 2^b$, we have the probability of a collision using b bits with m messages (sequences) is:

$$P(b, m) = m^2/2^{b+1}$$

Note that the collision probability depends on the number of messages, but not their size.

Solving for the number of messages (not used further in this analysis):

$$m(b, P) = \sqrt{P * 2^{b+1}}$$

Solving for the minimum number of *bits* b as a function of an expected number of sequences m and a desired tolerance for collisions of P:

$$b(m, P) = \log_2 \left(\frac{m^2}{P} \right) - 1$$

This equation is derived from equations that assume that $m \ll s$, where $s = 2^b$. When computing $b(m, P)$, we'll require that $m/s \leq 10^{-3}$ as follows:

$$m/s \leq 10^{-3}$$

is approximately equivalent to:

$$m/2^b \leq 2^{-5}$$

$$m \leq 2^{b-5}$$

$$log_2 m \leq b - 5$$

$$b \geq 5 + log_2 m$$

[5]:
```
def b2B3(b):
    """Convert bits b to Bytes, rounded up modulo 3

    We report modulo 3 because the intent will be to use Base64 encoding, which␣
↪is
    most efficient when inputs have a byte length modulo 3. (Otherwise, the␣
↪resulting
    string is padded with characters that provide no information.)

    """
    return math.ceil(b/8/3) * 3

def B(P, m):
    """return the number of bits needed to achieve a collision probability
    P for m messages

    Assumes m << 2^b.

    """
    b = math.log2(m**2 / P) - 1
    if b < 5 + math.log2(m):
        return "-"
    return b2B3(b)
```

[6]:
```
m_bins = [1E6, 1E9, 1E12, 1E15, 1E18, 1E21, 1E24, 1E30]
P_bins = [1E-30, 1E-27, 1E-24, 1E-21, 1E-18, 1E-15, 1E-12, 1E-9, 1E-6, 1E-3, 0.
↪5]
```

[7]:
```
table_rows = []
table_rows += [["#m"] + ["P<={P}".format(P=P) for P in P_bins]]
table_rows += [["-"] * len(table_rows[0])]
for n_m in m_bins:
    table_rows += [["{:g}".format(n_m)] + [B(P, n_m) for P in P_bins]]
table = "\n".join(["|".join(map(str,row)) for row in table_rows])
table_header = "### digest length (bytes) required for expected collision␣
↪probability $P$ over $m$ messages \n"
display(Markdown(table_header +  table))
```

1.4.1 digest length (bytes) required for expected collision probability P over m messages

#m	P<=1e-30	P<=1e-27	P<=1e-24	P<=1e-21	P<=1e-18	P<=1e-15	P<=1e-12	P<=1e-09	P<=1e-06	P<=0.001	P<=0.5
1e+06	18	18	15	15	15	12	12	9	9	9	6
1e+09	21	21	18	18	15	15	15	12	12	9	9
1e+12	24	24	21	21	18	18	15	15	15	12	12
1e+15	27	24	24	24	21	21	18	18	15	15	15
1e+18	30	27	27	24	24	24	21	21	18	18	15
1e+21	30	30	30	27	27	24	24	24	21	21	18
1e+24	33	33	30	30	30	27	27	24	24	24	21
1e+30	39	39	36	36	33	33	30	30	30	27	27

1.5 References

- [1] http://nvlpubs.nist.gov/nistpubs/FIPS/NIST.FIPS.180-4.pdf
- [2] https://tools.ietf.org/html/rfc3548#section-4
- [3] http://stackoverflow.com/a/4014407/342839
- [4] http://stackoverflow.com/a/22029380/342839
- [5] http://preshing.com/20110504/hash-collision-probabilities/
- [6] https://en.wikipedia.org/wiki/Birthday_problem
- [7] https://en.wikipedia.org/wiki/Birthday_attack
- [8] https://tools.ietf.org/html/rfc4648#section-5

1.6 Acknowledgements

Thanks to Bob Freimuth for identifying an error in a superscript that caused collision probabilities to be underestimated.

```
[ ]:
```
