# Supplementary material for: SeqRepo: A system for managing local collections of biological sequences
Source: PLoS One. 2020 Dec 3;15(12):e0239883. doi: 10.1371/journal.pone.0239883 (PMC7714221; doi:10.1371/journal.pone.0239883)
Supplement: S2 Code — (PDF) [file pone.0239883.s002.pdf]

# API Examples

September 13, 2020

## 0.1 Python library

[1]:
```python
from biocommons.seqrepo import SeqRepo
```

[2]:
```python
sr = SeqRepo("/usr/local/share/seqrepo/latest/")
```

[3]:
```python
# sequences may be fetched by a namespace alias
sr.fetch(namespace="GRCh38", alias="chr1", start=1000000, end=1000020)
```

[3]:
```
'GTGGAGCGCGCCGCCACGGA'
```

[4]:
```python
# SeqRepo also supports dictionary-style lookup
sr["refseq:NC_000001.11"][1000000:1000020]
```

[4]:
```
'GTGGAGCGCGCCGCCACGGA'
```

[5]:
```python
# aliases need to be namespace-qualified only if ambiguous
sr["NC_000001.11"][1000000:1000020]
```

[5]:
```
'GTGGAGCGCGCCGCCACGGA'
```

[6]:
```python
# non-unique aliases raise exceptions
try:
    sr["chr1"][1000000:1000020]
except KeyError as e:
    print(e)
```

```
'Alias chr1 (namespace: None): not unique'
```

[7]:
```python
sr.translate_alias("NC_000001.11")
```

[7]:
```
['GRCh38:1',
 'GRCh38:chr1',
 'GRCh38.p1:1',
 'GRCh38.p1:chr1',
 'GRCh38.p10:1',
 'GRCh38.p10:chr1',
 'GRCh38.p11:1',
```

```
 'GRCh38.p11:chr1',
 'GRCh38.p12:1',
 'GRCh38.p12:chr1',
 'GRCh38.p2:1',
 'GRCh38.p2:chr1',
 'GRCh38.p3:1',
 'GRCh38.p3:chr1',
 'GRCh38.p4:1',
 'GRCh38.p4:chr1',
 'GRCh38.p5:1',
 'GRCh38.p5:chr1',
 'GRCh38.p6:1',
 'GRCh38.p6:chr1',
 'GRCh38.p7:1',
 'GRCh38.p7:chr1',
 'GRCh38.p8:1',
 'GRCh38.p8:chr1',
 'GRCh38.p9:1',
 'GRCh38.p9:chr1',
 'MD5:6aef897c3d6ff0c78aff06ac189178dd',
 'NCBI:NC_000001.11',
 'refseq:NC_000001.11',
 'SEGUID:FCUd6VJ6uikS/VWLbhGdVmj2rOA',
 'SHA1:14251de9527aba2912fd558b6e119d5668f6ace0',
 'VMC:GS_Ya6Rs7DHhDeg7YaOSg1EoNi3U_nQ9SvO',
 'sha512t24u:Ya6Rs7DHhDeg7YaOSg1EoNi3U_nQ9SvO',
 'ga4gh:SQ.Ya6Rs7DHhDeg7YaOSg1EoNi3U_nQ9SvO']
```

---

### 0.1.1 SeqRepo REST Interface

[8]:
```
import requests
```

[9]:
```
requests.get("http://localhost:5000/seqrepo/1/ping").json()
```

[9]:
```
{'dependencies': {'bioutils': {'url': 'https://github.com/biocommons/bioutils/',
   'version': '0.5.2.post3'},
  'seqrepo': {'root': '/usr/local/share/seqrepo/latest',
   'url': 'https://github.com/biocommons/biocommons.seqrepo/',
   'version': '0.6.2'}},
 'url': 'https://github.com/biocommons/seqrepo-rest-service/',
 'version': '0.1.4.dev0+g832fedd.d20200905'}
```

[10]:
```
requests.get("http://localhost:5000/seqrepo/1/sequence/NC_000001.11?
 ↪start=100000&end=100020").text
```

[10]: 'ACTAAGCACACAGAGAATAA'

[11]:
```
requests.get("http://localhost:5000/seqrepo/1/metadata/NC_000001.11").json()
```

[11]:

```
{'added': '2016-08-27T21:17:00Z',
 'aliases': ['GRCh38:1',
  'GRCh38:chr1',
  'GRCh38.p1:1',
  'GRCh38.p1:chr1',
  'GRCh38.p10:1',
  'GRCh38.p10:chr1',
  'GRCh38.p11:1',
  'GRCh38.p11:chr1',
  'GRCh38.p12:1',
  'GRCh38.p12:chr1',
  'GRCh38.p2:1',
  'GRCh38.p2:chr1',
  'GRCh38.p3:1',
  'GRCh38.p3:chr1',
  'GRCh38.p4:1',
  'GRCh38.p4:chr1',
  'GRCh38.p5:1',
  'GRCh38.p5:chr1',
  'GRCh38.p6:1',
  'GRCh38.p6:chr1',
  'GRCh38.p7:1',
  'GRCh38.p7:chr1',
  'GRCh38.p8:1',
  'GRCh38.p8:chr1',
  'GRCh38.p9:1',
  'GRCh38.p9:chr1',
  'MD5:6aef897c3d6ff0c78aff06ac189178dd',
  'NCBI:NC_000001.11',
  'refseq:NC_000001.11',
  'SEGUID:FCUd6VJ6uikS/VWLbhGdVmj2rOA',
  'SHA1:14251de9527aba2912fd558b6e119d5668f6ace0',
  'VMC:GS_Ya6Rs7DHhDeg7YaOSg1EoNi3U_nQ9SvO',
  'sha512t24u:Ya6Rs7DHhDeg7YaOSg1EoNi3U_nQ9SvO',
  'ga4gh:SQ.Ya6Rs7DHhDeg7YaOSg1EoNi3U_nQ9SvO'],
 'alphabet': 'ACGMNRT',
 'length': 248956422}
```


---

### 0.1.2 refget API

[12]:
```python
requests.get("http://localhost:5000/refget/1/ping").json()
```

[12]:
```
'The secret is to bang the rocks together, guys.'
```

[13]:
```python
requests.get("http://localhost:5000/refget/1/sequence/service-info").json()
```

[13]:

```
{'service': {'algorithms': ['md5', 'trunc512'],
  'circular_supported': False,
  'subsequence_limit': None,
  'supported_api_versions': ['1.0']},
 'x-config': {'bioutils': {'url': 'https://github.com/biocommons/bioutils/',
   'version': '0.5.2.post3'},
  'seqrepo': {'root': '/usr/local/share/seqrepo/latest',
   'url': 'https://github.com/biocommons/biocommons.seqrepo/',
   'version': '0.6.2'},
  'seqrepo-rest-service': {'url': 'https://github.com/biocommons/seqrepo-rest-
service/',
   'version': '0.1.4.dev0+g832fedd.d20200905'}}}
```


[14]:
```python
requests.get("http://localhost:5000/refget/1/sequence/ga4gh%3ASQ.
↪Ya6Rs7DHhDeg7YaOSg1EoNi3U_nQ9SvO?start=100000&end=100020").text
```

[14]:
```
'ACTAAGCACACAGAGAATAA'
```

[15]:
```python
requests.get("http://localhost:5000/refget/1/sequence/ga4gh%3ASQ.
↪Ya6Rs7DHhDeg7YaOSg1EoNi3U_nQ9SvO/metadata").json()
```

[15]:

```
{'metadata': {'aliases': [{'alias': '1', 'naming_authority': 'GRCh38'},
   {'alias': 'chr1', 'naming_authority': 'GRCh38'},
   {'alias': '1', 'naming_authority': 'GRCh38.p1'},
   {'alias': 'chr1', 'naming_authority': 'GRCh38.p1'},
   {'alias': '1', 'naming_authority': 'GRCh38.p10'},
   {'alias': 'chr1', 'naming_authority': 'GRCh38.p10'},
   {'alias': '1', 'naming_authority': 'GRCh38.p11'},
   {'alias': 'chr1', 'naming_authority': 'GRCh38.p11'},
   {'alias': '1', 'naming_authority': 'GRCh38.p12'},
   {'alias': 'chr1', 'naming_authority': 'GRCh38.p12'},
   {'alias': '1', 'naming_authority': 'GRCh38.p2'},
   {'alias': 'chr1', 'naming_authority': 'GRCh38.p2'},
   {'alias': '1', 'naming_authority': 'GRCh38.p3'},
   {'alias': 'chr1', 'naming_authority': 'GRCh38.p3'},
   {'alias': '1', 'naming_authority': 'GRCh38.p4'},
   {'alias': 'chr1', 'naming_authority': 'GRCh38.p4'},
   {'alias': '1', 'naming_authority': 'GRCh38.p5'},
   {'alias': 'chr1', 'naming_authority': 'GRCh38.p5'},
```

```
 {'alias': '1', 'naming_authority': 'GRCh38.p6'},
 {'alias': 'chr1', 'naming_authority': 'GRCh38.p6'},
 {'alias': '1', 'naming_authority': 'GRCh38.p7'},
 {'alias': 'chr1', 'naming_authority': 'GRCh38.p7'},
 {'alias': '1', 'naming_authority': 'GRCh38.p8'},
 {'alias': 'chr1', 'naming_authority': 'GRCh38.p8'},
 {'alias': '1', 'naming_authority': 'GRCh38.p9'},
 {'alias': 'chr1', 'naming_authority': 'GRCh38.p9'},
 {'alias': '6aef897c3d6ff0c78aff06ac189178dd', 'naming_authority': 'MD5'},
 {'alias': 'NC_000001.11', 'naming_authority': 'NCBI'},
 {'alias': 'NC_000001.11', 'naming_authority': 'refseq'},
 {'alias': 'FCUd6VJ6uikS/VWLbhGdVmj2rOA', 'naming_authority': 'SEGUID'},
 {'alias': '14251de9527aba2912fd558b6e119d5668f6ace0',
  'naming_authority': 'SHA1'},
 {'alias': 'GS_Ya6Rs7DHhDeg7YaOSg1EoNi3U_nQ9SvO', 'naming_authority': 'VMC'},
 {'alias': 'Ya6Rs7DHhDeg7YaOSg1EoNi3U_nQ9SvO',
  'naming_authority': 'sha512t24u'},
 {'alias': 'SQ.Ya6Rs7DHhDeg7YaOSg1EoNi3U_nQ9SvO',
  'naming_authority': 'ga4gh'}],
'id': 'Ya6Rs7DHhDeg7YaOSg1EoNi3U_nQ9SvO',
'length': 248956422,
'md5': '6aef897c3d6ff0c78aff06ac189178dd',
'trunc512': '61ae91b3b0c78437a0ed868e4a0d44a0d8b753f9d0f52bce'}}
```

[ ]:
