# Supplementary material for: SeqRepo: A system for managing local collections of biological sequences
Source: PLoS One. 2020 Dec 3;15(12):e0239883. doi: 10.1371/journal.pone.0239883 (PMC7714221; doi:10.1371/journal.pone.0239883)
Supplement: S3 Code — (PDF) [file pone.0239883.s003.pdf]

Timings

September 13, 2020

1 Sequence Collection Timing Comparisons

This notebook compares timings for fetching short random genomic slices from GRCh38 using:

- SeqRepo native python interface (local sequences)
- SeqRepo REST interface (local sequences)
- ENA CRAM implementation of refget (the only public facility with human sequences at this time)
- NCBI E-utilities

The goal of these timings is to provide order-of-magnitude differences between the methods. No effort has been made to optimize any of these timings or to seek attain high-precision timings.

---

2 Fetch methods

For each of the above sources, a `fetch_<method>(accession, start, end)` method is defined below. Those methods are excuted using a single timing harness at the bottom of the notebook.

[ ]:
```
from base64 import urlsafe_b64decode, urlsafe_b64encode
from binascii import hexlify, unhexlify
import functools
import hashlib
import itertools
import json
import logging
import os
import random
import re
import time

import requests

from biocommons.seqrepo import SeqRepo
```

[ ]:
```
# https://stackoverflow.com/questions/48380452/
↪mask-out-sensitive-information-in-python-log
```

```python
_logger = logging.getLogger(__name__)

class RedactingFormatter(logging.Formatter):
    """Formatter that removes sensitive information in urls."""
    @staticmethod
    def _filter(s):
        return

    def format(self, record):
        s = logging.Formatter.format(self, record)
        s = re.sub(r"(ncbi_api_key|ncbi_api_tool|ncbi_api_email)=[^&]+",␣
 ↪r"\1=…", s)
        return s

for handler in _logger.root.handlers:
    handler.setFormatter(RedactingFormatter(handler.formatter._fmt))
```

setup fetch_seqrepo_python()

[ ]:
```python
sr = SeqRepo("/usr/local/share/seqrepo/latest")

def fetch_seqrepo_python(accession, start=None, end=None):
    return sr.fetch(accession, start, end)
```

fetch_seqrepo_rest()

[ ]:
```python
seqrepo_base_url = "http://localhost:5000/seqrepo/1/"
seqrepo_session = requests.Session()
seqrepo_session.request = functools.partial(seqrepo_session.request, timeout=2.
 ↪0)

def fetch_seqrepo_rest(accession, start=None, end=None):
    url = seqrepo_base_url + "sequence/" + accession
    params = {
        "start": start,
        "end": end
    }
    resp = seqrepo_session.get(url, params=params)
    resp.raise_for_status()
    return resp.text
```

setup fetch_refget_ena()

[ ]:
```python
refget_session = requests.Session()
refget_session.request = functools.partial(refget_session.request, timeout=2.0)
refget_session.params.update({
    "accept": "text/plain"
```

```
})

refget_base_url = "https://www.ebi.ac.uk/ena/cram/sequence"

def fetch_refget_ena(md5, start=None, end=None):
    url = refget_base_url + "/" + md5
    params = {}
    if start:
        params["start"] = start
    if end:
        params["end"] = end
    resp = refget_session.get(url, params=params)
    resp.raise_for_status()
    return resp.text
```

fetch_ncbi_eutils() Get an NCBI API key at https://www.ncbi.nlm.nih.gov/account/settings/, then create `~/.config/ncbi.json` with this template:

```
{
  "ncbi_api_key": "3a5...910",
  "ncbi_api_tool": "my tool",
  "ncbi_api_email": "myemail"
}
```

In practice, NCBI services exhibit sporadic errors that succeed with immediate retry and appear to be unrelated to rate limiting. Therefore, this function is more complicated than the others because it attempts to provide rate limiting and retry logic.

[ ]:
```
ncbi_credentials_fn = os.path.expanduser("~/.credentials/ncbi.json")
ncbi_credentials = json.load(open(ncbi_credentials_fn))

efetch_url = "https://eutils.ncbi.nlm.nih.gov/entrez/eutils/efetch.fcgi"
rate_limit = 10                        # queries/sec
query_period = 1.0/rate_limit          # period between queries

ncbi_session = requests.Session()
ncbi_session.request = functools.partial(ncbi_session.request, timeout=2.0)
ncbi_session.params.update({
    "db": "nucleotide",
    "rettype": "fasta",
    "retmode": "text"})
ncbi_session.params.update(ncbi_credentials)

from requests.adapters import HTTPAdapter
ncbi_session.mount("https://", HTTPAdapter(max_retries=3))

ncbi_last_query_time = 0
def fetch_ncbi_eutils(accession, start=None, end=None):
```

```
    global ncbi_last_query_time
    params = {"id": accession}
    if start:
        params["seq_start"] = start + 1
    if end:
        params["seq_stop"] = end
    time_now = time.time()
    sleep_time = max(0, ncbi_last_query_time + query_period - time_now)
    time.sleep(sleep_time)
    ncbi_last_query_time = time_now
    resp = ncbi_session.get(efetch_url, params=params)
    resp.raise_for_status()
    fasta = resp.text
    return fasta[fasta.find("\n")+1:].replace("\n","")
```

3 Build sequence slice expressions

The goal is to build a list of sequence slices – short random excerpts of GRCh38. All fetch methods will be called with the same slices. (ENA refget understands only md5 sequence identifiers, so the refseq accessions are translated ahead of time.)

[ ]:
```
# acs: list of grch38 primary accession refseq ids
acs = [
    'NC_000001.11', 'NC_000002.12', 'NC_000003.12', 'NC_000004.12', 'NC_000005.
↪10',
    'NC_000006.12', 'NC_000007.14', 'NC_000008.11', 'NC_000009.12', 'NC_000010.
↪11',
    'NC_000011.10', 'NC_000012.12', 'NC_000013.11', 'NC_000014.9',  'NC_000015.
↪10',
    'NC_000016.10', 'NC_000017.11', 'NC_000018.10', 'NC_000019.10', 'NC_000020.
↪11',
    'NC_000021.9',  'NC_000022.11', 'NC_000023.11', 'NC_000024.10']

# ac_lengths = {refseq_ac: sequence length} (from SeqRepo metadata)
ac_lengths = {ac: len(sr[ac]) for ac in acs}

def lookup_md5(sr, ac):
    s = [a for a in sr[ac].aliases if a.startswith("MD5:")][0]
    return s[4:]

# ac_md5s = {refseq_ac: md5} (from SeqRepo aliases)
ac_md5s = {ac: lookup_md5(sr, ac) for ac in acs}

# build two sets of equivalent slices, one with refseq accession, the other␣
↪with md5 (for refget)
def random1():
```

```
    max_size = 25
    ac = random.choice(acs)
    start = random.randint(0, ac_lengths[ac] - max_size)
    end = start + random.randint(1, 20)
    return (ac, start, end)
```

4 Timing

[ ]:
```
import collections
from requests.exceptions import RequestException

def time1(fx, slices):
    """execute fx on each s in slices, returning tuple of (elapsed time,␣
 ↪n_exceptions)

    elapsed time (rather than cpu time) is used because elapsed time is a
    better proxy for user experience

    """

    def exec1(fx, s):
        try:
            return fx(*s)
        except (RequestException) as e:
            return e

    t0 = time.time()
    res = [exec1(fx, s) for s in slices]
    tdelta = time.time() - t0
    errors = [r for r in res if isinstance(r, Exception)]
    error_counts = collections.Counter(type(e) for e in errors)
    return {
        "etime": tdelta,
        "errors": error_counts.most_common(),
        "n_errors": len(errors),
        "throughput": len(slices)/tdelta
        }


def time_all(n_slices):
    refseq_slices = [random1() for _ in range(n_slices)]
    md5_slices = [(ac_md5s[rs[0]], rs[1], rs[2]) for rs in refseq_slices]
    results = {
        "ncbi_eutils": time1(fetch_ncbi_eutils, refseq_slices),
        "refget_ena": time1(fetch_refget_ena, md5_slices),
        "seqrepo_python": time1(fetch_seqrepo_python, refseq_slices),
```

```
        "seqrepo_rest": time1(fetch_seqrepo_rest, refseq_slices)
        }
    return results
```

[ ]:
```
# smoke test: fetch 1 random sequence slice
time_all(1)
```

[ ]:
```
# run timing tests on 1000 random sequence slices
# N.B. The timings reported in the manuscript were generated on two␣
↪environments:
# NCBI E-utilities and ENA refget timings were generated on a c4.large instance␣
↪in AWS
# SeqRepo Python and REST timings were generated on a laptop with SSD
# time_all(1000)
```
